# Waste to Energy: Combustion, Performance, and Emission Characteristics of Waste Animal Fats/Diesel Blends Premixed with Various Alcohols as Port Fuels

**DOI:** 10.1002/gch2.202400189

**Published:** 2024-10-13

**Authors:** T. Sathish, Jayant Giri, R. Saravanan, Ümit Ağbulut

**Affiliations:** ^1^ Department of Mechanical Engineering Saveetha School of Engineering, SIMATS Chennai Tamil Nadu 602105 India; ^2^ Division of Research and Development Lovely Professional University Phagwara India; ^3^ Department of Mechanical Engineering Yeshwantrao Chavan College of Engineering Nagpur Maharashtra 441110 India; ^4^ Department of Mechanical Engineering Mechanical Engineering Faculty Yildiz Technical University Istanbul 34349 Türkiye; ^5^ Department of Technical Sciences Western Caspian University Baku AZ1001 Azerbaijan; ^6^ Department of Chemistry and Biochemistry, California State University, Los Angeles State University Drive Los Angeles CA 515190032 USA

**Keywords:** chicken fat biodiesel, combustion, port Fuels, waste management, waste to energy

## Abstract

Animal flesh is a major food source with economic and industrial value for consumer demand. These meats produced biowaste during and after preparation and use. Chicken intestines make up most of the waste thrown away after processing or frying. This study considers it a biodiesel source. Transesterification turns chicken intestine waste fat oil into biodiesel. This oil is used in compression ignition (CI) engines but performs poorly compared to diesel. Diesel, the base fuel, is mixed with 20% biodiesel. The remaining 10% and 20% of butanol and pentanol are port fuels, improving combustion and lowering emissions in the 5.2 kW, 1500 rpm CI engine. 20% pentanol premixing outperformed butanol premixing, blending, and engine CIWFOB operation. The greater heating value improves combustion, therefore 20% pentanol premixing with blend produces 32.76% BTE, 10.57% more than diesel. It produced 55.18% less CO and 50.92% less smoke than diesel, which has a greater heat release rate (48.86 J/CAD) and peak pressure (64.76 bar). This premixing costs NOx emissions. The CIWFOB blend with 20% pentanol premixing improves engine performance. For SDGs 7, 9, 12, and 13, this study is supported.

## Graphical abstract

Waste animal fat is converted into alternative fuels for compression ignition engines, and the results regarding engine performance, combustion, and emission characteristics are discussed. The present research helps waste management and the usability of novel fuels as fuel substitutes. Also, this work supports attaining the Sustainable Development Goals (SDGs) of 7, 9, 12, and 13.

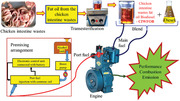

## 1 Introduction

In recent decades, there has been an increase in concern over the depletion of fossil fuels and their detrimental effects on both the environment and human health. When fossil fuels are continuously used, toxic gases like carbon dioxide, carbon monoxide, and nitric oxide are released into the atmosphere. These are the main greenhouse gas pollutants that have an impact on our ecosystem^[^ ^1^ ^]^ These fossil fuels are used so extensively in industry and transportation that it is impossible to ignore them.^[^ ^2^, ^3^ ^]^

Supporting fuels like biodiesel,^[^ ^4^ ^]^ alcohols,^[^ ^5^ ^]^ and various enhanced combustion techniques^[^ ^6^ ^]^ are to be developed further, which could potentially decrease the consumption of fossil fuels.^[^ ^7^ ^]^ Various researchers are concentrating on their studies about the availability of sources in the nation, such as its agricultural background, with regard to the production of biodiesel. The majority of bio‐diesels are derived from bio‐resources such as cotton, rapeseed, mustard oil, palm, sunflower, neem, and jatropha.^[^ ^8^, ^9^, ^10^, ^11^, ^12^ ^]^ These sources are facing cultivation, environment, and availability issues while using.^[^ ^12^ ^]^

Sources of biodiesel from waste are a recent trend in research. Because though wastes are biodegradable, the conversion of that waste into useful products supports environmental pollution.^[^ ^43^ ^]^ The waste‐to‐product transformation procedure allows for nearly full product utilization. Researchers simultaneously concentrated on identifying the waste and the technology needed to convert waste into usefulness.^[^ ^44^ ^]^

Different wastes such as cooked or fried oil^[^ ^13^ ^]^ waste from Hotels and Hostels,^[^ ^14^ ^]^ chicken waste from Meat shops/ food processing industries, mutton waste from the Meat shop/food processing industries, Pork shop waste from pork shops,^[^ ^15^ ^]^ and fish waste from the fish market^[^ ^16^, ^17^ ^]^ can be collected to produce the alternate fuel^[^ ^18^ ^]^ for diesel engines. These are common Meat wastes and also other non‐bio‐wastes that were tried to produce fuels for diesel engines.^[^ ^19^, ^20^ ^]^

Using methyl esters derived from chicken waste and a calcium oxide catalyst made from discarded eggshells, they tested the fuel at the constant speed of 3000 rpm.^[^ ^21^ ^]^ The study examined the effects of diesel blends with different proportions of biodiesel at different loads. The eggshell catalyst was used to produce biodiesel. Compared to diesel, the 30% biodiesel blend emitted fewer harmful pollutants such as emissions of carbon monoxide (CO), hydrocarbons (HC) at 20 ppm, and nitrogen oxide (NOx) at 50 ppm.^[^ ^22^ ^]^ Waste chicken oil, waste tire oil, and waste cooking oil 10% each oil blended with 70% diesel. The blend was tested at a constant speed of 2500 rpm, in a 4.9 kW CI engine.^[^ ^45^ ^]^ Here, the scraped chicken fat was used. This chicken waste oil has 40 MJ kg^−1^ of calorific value, 4.11 cSt of viscosity, and a cetane number of 56. At peak load, this chicken waste biodiesel blend has 380 g kWh^−1^ of brake‐specific fuel consumption, 190 K of exhaust gas temperature, 0.025% of CO emission, and 452 ppm of NOx. These all are less than diesel except brake‐specific fuel consumption.^[^ ^21^ ^]^

Waste chicken oil, waste tire oil, and waste cooking oil 10% each oil blended with 70% diesel. The blend was tested at a constant speed The effect of adding anisole to waste cooking oil methyl ester on the combustion, emission, and performance characteristics of a diesel internal combustion engine that has not been modified in any way.^[^ ^48^ ^]^ Through the utilization of response surface methodology, it was possible to make a prediction regarding the emissions and performance of a diesel engine that was powered by waste cooking oil and C8 oxygenate blends were also tested.^[^ ^49^ ^]^

Diesel and butanol (0% to 40%) blend was used to optimize their parameters like injection pressure of the fuel, injection stating position, compression ratio, and the percentage of the exhaust gas recirculation by response surface methodology through the NOx and soot emission with fuel consumption. Increasing butanol blending reduces the NOx emission from 8.04 g kWh^−1^ to 6.9 g kWh^−1^, soot from 1.44 to 0.855 g kWh^−1^, but increases the cylinder pressure from 58.83 to 59.49 bar, also increase of fuel consumption from 205 to 223 g kWh^−1^.^[^ ^23^ ^]^

An IC engine operates with the mahua biodiesel as the main fuel and the butanol as secondary fuel by supplying them in the main injection and port injection respectively. The butanol energy share varied from zero to 30% of the total energy share supplied by the mahua biodiesel to the engine as a heat supply. Here, the maximum energy share of the butanol produced the lesser BTE, and least NOx emission of 164 ppm, CO emission of 0.28% with higher peak pressure and heat release rate. However, 20% energy share produced a maximum BTE of 31.7% and a lesser smoke opacity of 41.4%.^[^ ^24^ ^]^ An investigation into the emission, engine performance, and combustion characteristics, of a diesel engine that was fueled with blends of Prunus domestica methyl ester and n‐butanol was carried out by.^[^ ^46^ ^]^ The 5.2 kW engine operated with the dual fuel mode with the main fuel of the tire oil as a 20% blend with diesel and nano fuel of tire oil bend created with the copper and zinc oxide nanoparticles. The secondary fuel is 10 to 30% of butanol. The operation is done on different loads. The peak BTE of 29.25% is achieved by the nano fuel but it has higher smoke and NOx emissions than the blend and dual fuel operation. But 30% premixing of butanol with the nano fuel has produced 8% higher BTE, as well as 23%, and 12% lesser smoke, and NOx emission than the base fuel.^[^ ^25^ ^]^ This study examined the effects of acetone‐butanol‐ethanol (ABE) diesel blends in an HCCI‐DI engine. It found that while the addition of ABE successfully decreased CO_2_ and NOx emissions, it did not reduce PM and soot emissions in an experimental investigation on an HCCI‐DI engine fueled with ABE. Additionally, higher BTE, lower BSFC, and EGT were observed, indicating excellent engine performance.^[^ ^36^ ^]^ Test fuel for a conventional engine was a blend of Mahua biodiesel and n‐butanol. A mixture of 20–30 vol% n‐butanol and 80 vol% diesel was tested. There was a range of 21 °CA bTDC to 25 °CA bTDC for the injection timing. In comparison to diesel fuel, the nitrogen oxide and carbon monoxide levels of a blend of B20 + D80 + 30% n‐butanol (NBM blend) at 21 °CA bTDC were lowered by 49% and 5.88%, respectively. At 25 °CA bTDC, the NBM blend's hydrocarbon and smoke emissions are reduced by 38.07% and 40%, respectively, in comparison to diesel. In comparison to all other test blends, the NBM blend's brake thermal efficiency was found to be higher at all injection timings. When comparing the NBM blend to diesel fuel, the brake thermal efficiency at 25 °CA bTDC increased by 15.30%.^[^ ^37^ ^]^

Possible increases in BTE and fuel efficiency were noted when butanol was added to diesel fuel blends. Using butanol as a biofuel significantly lowered CO emissions and exhaust gas temperatures, indicating the potential of this specific biofuel. On the other hand, increases in smoke opacity and HC emissions were noted, indicating the difficulties in utilizing biofuels like butanol. When predicting engine performance parameters using butanol as a biofuel, the cascade neural network demonstrated exceptional accuracy. Overall, the study provides insightful information about butanol's potential uses as a biofuel as well as its difficulties and obstacles, highlighting the significance of ongoing research into sustainable biofuels like butanol.^[^ ^38^ ^]^

Response Surface Methodology (RSM) and Artificial Neural Network (ANN) techniques were used to model the optimal production yield of Food Industrial Waste Oil Methyl Ester (FIWOME, B100, FIWOB). With a methanol/oil molar ratio of 5.99, a KOH content of 1.1 wt.%, and a duration of the reaction was 77.6 min, the highest yield of FIWOME of 92.5% was attained.^[^ ^39^ ^]^ Biodiesel characteristics, transesterification, waste materials, and policies promoting biodiesel production from waste were all studied using computational and machine‐learning techniques. Catalyst design and deactivation, reaction and reactor optimization, stability assessment, waste feedstock analysis, process scale‐up, reaction mechanics, and molecular dynamics simulation are all done using computational techniques. Waste feedstock includes cooking oil, animal fat, vegetable oil, algae, fish waste, municipal solid waste, and sewage sludge.^[^ ^40^, ^41^ ^]^ proposed developments in low‐energy catalytic processes that are relevant to the study of optimizing biodiesel production using sophisticated algorithms and heterogeneous catalysts, including CO_2_ capture, biodiesel production, and enzyme mimicking. Adding this will highlight the significance of your work within the larger field of sustainable chemical production and provide insightful context. It thoroughly discusses the wider ramifications of low‐energy catalytic processes in the production of sustainable chemicals, which is in line with your work's goal of producing biodiesel as efficiently as possible. In particular, the talks about catalyst optimization and the possibility of major energy efficiency gains could offer a strong theoretical basis for your biodiesel production research. With an emphasis on catalyst synthesis, structure, and applications,^[^ ^42^ ^]^ reviewed environmental photocatalysis, biocatalysis, and electrocatalysis. Metal‐organic frameworks, nanoparticles of non‐noble metals, nanocomposites, biomass‐derived materials, and enzymes are examples of common catalysts. By using thermogravimetry, photoelectron spectroscopy, X‐ray diffraction, and the Brunauer‐Emmett‐Teller isotherm, structures are characterized. According to our research, heterogeneous Fenton catalysis is one method that can degrade water pollutants with an efficiency ranging from 71.7% to 100%. With a generation rate exceeding 100 µmol h^−1^, dihydrogen (H_2_) was produced via photocatalysis. Following methane cracking, dihydrogen yields varied from 27% to 88%. Producing 48.6% to 99% biodiesel was achieved. Blends of methanol and diesel as well as the effects of engine loads on engine performance and tailpipe emissions. With increasing methanol blends, NOx has gone up. In contrast, CO emissions were decreased when HCs were added in greater proportion to methanol. Additionally, adding more methanol to methanol/diesel blends resulted in a significant reduction in smoke emissions.^[^ ^46^ ^]^

There is an extensive amount of chicken produced worldwide; estimates put the total annual production of chicken meat at over 137 million metric tonnes. The increasing demand for chicken as a main source of protein in different parts of the world is reflected in this high level of production.^[^ ^21^ ^]^ Globally, the average annual per capita consumption of chicken is ≈17 kg. Regional variations result in significantly different average consumption rates; the United States, for example, has far higher average consumption rates, averaging ≈50 kg per person annually.^[^ ^22^ ^]^ Feathers, bones, blood, and intestines are among the many waste products produced by the chicken processing industry. It is estimated that between 20% and 25% of the weight of the chicken is wasted. This equates to millions of units based on global production figures.^[^ ^45^ ^]^

In chicken shops, intestine waste is unavoidable as they cannot be sold. So, they dump it in a landfill. Though they are biodegradable, they are causing severe environmental pollution. The biodiesel is derived from such waste through transesterification called Chicken Intestine Waste Fat Oil Biodiesel (CIWFOB). The neat CIWFOB can be used in diesel engines, but its performance will be poorer than diesel's performance. Hence this biodiesel must be blended with suitable additives to achieve equivalent performance to diesel. This investigation aims to use novelty in preparing blends with a premix of butanol (10%, and 20%) and Pentanol (10% and 20%) separately to improve the results of the combustion, consumption, and emissions performance than the diesel and base fuel. The prepared blends will be Characterized and tested in the IC engine test rig.

This study presents a novel method for producing biodiesel by using the underutilized and frequently removed byproduct of the chicken industry. The waste from the chicken namely intestinal waste considers as a feedstock for the process. This study presents an innovative blend of 20% chicken intestine waste fat oil biodiesel (CIWFOB) and 80% diesel, with different amounts of butanol and pentanol added as port fuels. The findings show that when pentanol is used at 20% premixing, brake thermal efficiency (BTE) is significantly increased and harmful emissions like CO and smoke are significantly reduced. This provides a cost‐effective and environmentally friendly substitute for conventional diesel. This research is at the forefront of sustainable energy solutions and waste management strategies due to the trade‐off observed with NOx emissions, which offers valuable insights for further optimization.

## 2 Experimental Section

### 2.1 Fat Oil Collection from the Chicken Intestines

In chicken meat shops major physical parts of the chicken were utilized. Mostly the intestines were thrown as waste in the chicken meat shops. These intestines were collected from the meat shop. In 80 to 100 kg chicken meat selling shops produced 10 to 15 kg of intestines wastes on average. These intestines were collected, and an equal amount of water was added and heated up to 70 °C. Water was added to aid in the separation of fat from other intestinal constituents. Due to its lower density, fat floats on top of the non‐fatty sections of the intestines, which were suspended or dissolved in water. Water helps break down the tissues, increasing the amount of fat that can be extracted. In the transesterification process, which turns fat into biodiesel, this was crucial. The typical melting point of animal fats, such as those present in the intestines of chickens, was between thirty and forty degrees Celsius. Melting the fat, which has a melting point near this temperature, was aided by heating the mixture to 70 °C. Once melted, the fat makes it easier to separate from other tissue constituents and water. Additionally, heating contributes to the reduction of the microbial load, which sterilizes the waste material prior to processing and was necessary for safe handling. In the intestines, proteins and other water‐soluble substances start to denature and dissolve in water at 70 °C, which facilitates the separation process. The fat oil floats on the water's surface. This fat oil was collected continuously. The skimming method employed in the collection of melted fat oil that was the fat was gently scooped off the surface of the water. Up until the fat oil float stops or neglected quantity of fat oil float, keep doing this process. 1000 mL of chicken intestinal fat oil was produced from 8.5 kg of chicken intestinal waste.

### 2.2 Chicken Intestine Waste fat Oil Biodiesel

**Figure 1:**
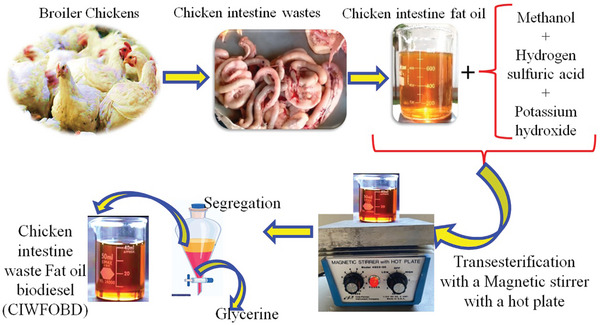
Schematic of transesterification of biodiesel production from chicken intestine wastes.

During the transesterification process, the chicken intestine fat oil that had been collected was utilized in the production of biodiesel. The quantity of chicken intestine fat oil that was taken was blended with one‐fourth of the methyl alcohol that was taken together. In addition, 5 mg L^−1^ of hydrogen sulphuric acid was added as the acid catalyst. the blending was carried out in a magnetic stirrer at a speed of 300 rpm for 60 min at a temperature of 60 °C. After that 5 mg L^−1^ of potassium hydroxide (KOH) was mixed as the acid catalyst for the higher biodiesel yield. The transesterification reaction, which was the chemical reaction between the triglycerides in the fat and methanol, was enhanced by the presence of potassium hydroxide, which acts as a catalyst and speeds up the process. By means of this reaction, the triglycerides were transformed into methyl esters, which were biodiesel, as well as glycerol. To achieve a balance between complete reaction and avoiding excess catalysts that could lead to soap formation or other side reactions, which could potentially lower the quality and yield of the biodiesel, the amount of KOH that was added was carefully optimized to be 5 mg L^−1^ in this particular instance. In the case of feedstocks that contain a high amount of free fatty acids (FFA), such as animal fats, KOH helps in neutralizing these FFAs, which, if left unchecked, have the potential to form soap and impede the process of transesterification. For the purpose of achieving a higher yield of biodiesel, this neutralization process was absolutely necessary. Triglycerides were converted into biodiesel at a faster rate when KOH was added to the mixture. Without an efficient catalyst, the reaction would proceed at a glacial pace, which would result in a reduction in the amount of biodiesel produced. After that, the same condition of temperature and speed of the stirrer was maintained for 180 min. The biodiesel had appeared. Then this mixture was placed for a day in the inverted conical flask to separate the biodiesel and settled glycerine. In the process of producing biodiesel, particularly during the cleaning or washing stages, water was frequently utilized to facilitate the elimination of impurities and contaminants. There was a possibility that any residual water that was left in the biodiesel could result in many problems, including a decrease in the fuel's efficiency and the possibility of corrosion occurring in the components of the engine. The biodiesel was heated to a temperature of 100 °C, which was higher than the boiling point of water (100 °C at standard atmospheric pressure). This causes any water that was still present in the form of vapor to evaporate and be driven off, leaving behind only pure biodiesel fuel. To ensure complete water removal, a drying agent of silica gel was used after the heating process. This agent helps to absorb any remaining water, ensuring the biodiesel was dry and suitable for used in engines. The mixture was subjected to transesterification using a magnetic stirrer with a hot plate to ensure thorough mixing and proper reaction conditions. During this reaction, the triglycerides in the fat oil react with methanol to form methyl esters (biodiesel) and glycerine. After the transesterification reaction, the mixture was allowed to settle, leading to the separation of biodiesel (CIWFOB) from glycerine. The two layers were segregated, with glycerine being removed as a byproduct with the use of a separating funnel. Chicken intestinal waste fat oil biodiesel (CIWFOBD) was the name given to this biodiesel. The composition of Chicken Intestine Waste Fat Oil Biodiesel (CIWFOBD) consists of fatty acid methyl esters (FAME), which were the primary components formed during the transesterification of chicken waste fats. The palmitic acid (C16:0) and stearic acid (C18:0) of Saturated fatty acids (SFA) were 27% and 7% respectively. Monounsaturated fatty acids (MUFA) / oleic acid (C18:1), was 43%. Polyunsaturated Fatty Acids (PUFA) / Linoleic Acid (C18:2) was 16%. **Figure** **1** discusses the entire procedure.

Figure 1 gives a schematic view of the transesterification of biodiesel production from chicken intestine wastes. Waste from chicken intestines was first gathered and processed to remove fat, which was then used as the main source of oil for making biodiesel. Methanol was utilized as the alcohol component in a transesterification reaction that was performed on the extracted oil. Hydrogen sulfuric acid and potassium hydroxide were combined to catalyze the reaction. These catalysts made it easier for the triglycerides in fish and chicken fat oils to be converted into methyl esters, which are the building blocks of biodiesel, and glycerine as a byproduct. A magnetic stirrer equipped with a hot plate was used to continuously stir the reaction mixture, enabling a uniform mixture and maintaining the temperature needed for the reaction to proceed effectively. After the reaction was finished, the mixture was allowed to settle, which caused the glycerine and biodiesel to separate. After sinking to the bottom, the denser glycerine was separated from the biodiesel. After the separation process, the finished product known as Chicken Intestine Waste Fat Oil Biodiesel, or CIFFOBD was obtained successfully. With the help of this process, waste materials of chicken intestines could be effectively converted into biodiesel, supporting the management of waste and facilitating the development of renewable energy.

### 2.3 Investigation Flow

**Figure 2:**
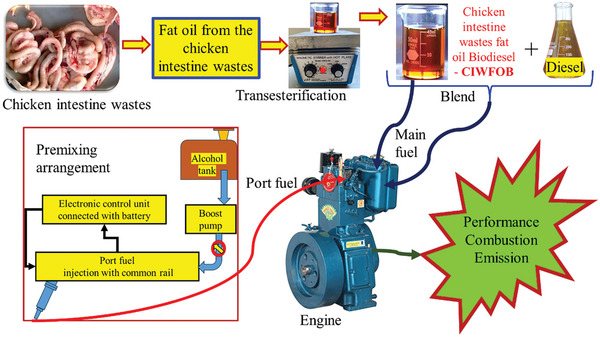
CIWFOB production and testing approach with Butanol/ Pentanol premixed conditions.

The test fuels used for the investigations were mentioned in **Table** **1** and the flow of work was mentioned in **Figure** **2**. The collection of chicken intestinal waste and its subsequent processing to extract fat oil represented the start of the experimental process for making Chicken Intestine Waste Fat Oil Biodiesel (CIWFOBD). Using a magnetic stirrer and a hot plate, this extracted fat oil was subsequently put through a transesterification process that produced CIWFOBD. Afterward, different fuel blends were created by blending the CIWFOBD with neat diesel.

**Table 1:** Composition of test fuels with and without Butanol/Pentanol premixing conditions.

Fuel Index	Fuel/Fuel Blend	Fuel content		Condition of Intake Air
		Diesel	CIWFOBD	
100D	Neat Diesel	100%	0	Fresh Air
CIWFOBD	Neat Biodiesel	0	100%	Fresh Air
80D+20CIWFOBD	B20 Grade Biodiesel	80%	20%	Fresh Air
80D+20CIWFOBD‐Bu10%	B20 Grade Biodiesel	80%	20%	Fresh Air +10% Butanol
80D+20CIWFOBD‐Bu20%	B20 Grade Biodiesel	80%	20%	Fresh Air +20% Butanol
80D+20CIWFOBD‐Pen10%	B20 Grade Biodiesel	80%	20%	Fresh Air +10% Penanol
80D+20CIWFOBD‐Pen20%	B20 Grade Biodiesel	80%	20%	Fresh Air +20% Penanol

In particular, the fuel blends were prepared such as (listed in Table 1) A mixture of 80% Diesel and 20% CIWFOBD was known as 80D+20CIWFOBD. A mixture consisting of 80% Diesel, 20% CIWFOBD, and 10% Butanol. A mixture of 80% Diesel, 20% CIWFOBD, and 20% Butanol was known as 80D+20CIWFOBD + Butanol 20%. A mixture of 80% Diesel, 20% CIWFOBD, and 10% Pentanol was known as 80D+20CIWFOBD + Pentanol 10% A mixture of 80% Diesel, 20% CIWFOBD, and 20% Pentanol was known as 80D+20CIWFOBD + Pentanol 20%. The performances in terms of combustion, engine, and emission characteristics of these six fuel blends were then examined in a Direct Injection (DI) diesel engine test rig in conjunction with neat diesel. In order to evaluate how well the fuel blends burned during testing, the In‐Cylinder Pressure (ICP) and Heat Release Rate (HRR) were monitored.

By examining the Brake Thermal Efficiency (BTE) and Brake Specific Fuel Consumption (BSFC), the engine's performance was assessed. To compare the environmental impact of each fuel blend, additional emission characteristics were measured, such as Carbon Monoxide (CO), Smoke Opacity, Nitrogen Oxides (NOx), and Unburned Hydrocarbons (HC). The optimal blend for use in diesel engines was then determined by comparing the test results, considering both emission and performance factors. Hence, there were seven kinds of fuels tested under the same conditions in the CI engine test rig. Before they were tested in diesel engines, they were characterized for fuel properties including the neat diesel. The properties of the fuels were tabulated in **Table** **2**. It must be noted that the fuel blend B20 (20% biodiesel +80% diesel) was chosen based on the trial experiments. Similarly, the choice of butanol (10%, and 20%) and Pentanol (10% and 20%) was also made through trial experiments. The following were the merits of considering alcohol. Pentanol and butanol are both examples of alcohols that have oxygen‐containing molecules within their molecular structures. Alcohols like these were added to biodiesel, which results in an increase in the overall oxygen content of the fuel blend.^[^ ^4^ ^]^ The presence of this additional oxygen encourages a more complete combustion, which in turn reduces the production of incomplete combustion products such as carbon monoxide (CO) and hydrocarbons that have not been burned over.^[^ ^5^, ^25^ ^]^ When compared to biodiesel, butanol and pentanol have lower auto‐ignition temperatures, which results in a shorter ignition delay. Consequently, this contributes to the achievement of a combustion process that is more controlled and efficient, thereby enhancing the brake thermal efficiency (BTE) of the engine.^[^ ^28^ ^]^ As a result of the addition of butanol and pentanol to the fuel blend, the oxygen availability in the fuel blend has increased, which contributes to the more complete oxidation of carbon‐based intermediates, which in turn reduces emissions of carbon monoxide and hydrocarbons.^[^ ^29^, ^30^ ^]^ Butanol and pentanol with a higher latent heat of vaporization have the potential to produce a cooling effect during the intake and compression strokes of the combustion process.^[^ ^35^ ^]^ This has the potential to reduce the peak temperatures inside the cylinders, which could therefore lead to a reduction in the production of nitrogen oxides (NOx).^[^ ^23^ ^]^ However, the impact on NOx may vary depending on the conditions of the engine. In addition, the presence of alcohol in the fuel blend was a factor that contributes to a decrease in the color of the smoke.^[^ ^24^ ^]^ The reason for this was that the improved atomization and evaporation of alcohols results in a more homogenous mixture of air and fuel, which in turn reduces the formation of soot.^[^ ^27^ ^]^

**Table 2:** Physicochemical properties of test fuels.

Properties	Unit	D100	CIWFOBD	80D+20CIWFOBD	Butanol	Pentanol
Cetane number	–	50	44	48	17	20
Calorific value	kJ/kg	42 450	36 880	41 336	32 650	34 650
Density	kg/m^3^	821	854	830	809	816
Flash point	°C	69	93	74	36	48
Heat of vaporization	kJ/kg	284	394	320	596	310
Oxygen content	%	0	14.25%	5.20%	21.00%	18.00%
Viscosity	cSt	2.84	3.51	2.97	2.7	2.9

It is possible for the cold flow properties of biodiesel blends to be improved through the addition of butanol and pentanol.^[^ ^37^ ^]^ It is common for biodiesel to have higher pour and cloud points, which can be problematic in instances where the temperature is low.^[^ ^38^ ^]^ These temperatures were lowered by the alcohols, which makes the biodiesel blend more suitable for use in environments with a lower average temperature.^[^ ^47^ ^]^ However, butanol and pentanol were miscible with both diesel and biodiesel, which guarantees that the mixture will be homogenous and will not separate into phases.^[^ ^29^ ^]^ Because of this, the fuel blend will be more stable over the course of time.^[^ ^27^ ^]^ It is also possible for the presence of alcohols to assist in lowering the oxidation tendency of biodiesel, which is more susceptible to degradation as a result of the presence of unsaturated fatty acids in it. Both the biodiesel blend's shelf life and its usability are increased as a result of this.^[^ ^37^ ^]^

#### 2.3.1 Cetane Number

The ignition of a fuel could be measured by its cetane number. Shorter ignition delays were indicated by higher cetane numbers, which promote smoother and more effective combustion. Because diesel (D100) had the highest cetane number (50), combustion was more efficient and there was less ignition delay. Its comparatively high Brake Thermal Efficiency (BTE) and lower emissions when compared to CIWFOBD were a result of this. Compared to diesel, CIWFOBD has a lower cetane number (44) which denotes a longer ignition delay, poorer combustion efficiency, and higher emissions. When CIWFOBD was blended with diesel, the cetane number (48) is raised, which shortens the ignition delay when compared to CIWFOBD alone. This improves combustion and lowers emissions. The cetane numbers of butanol (17) and pentanol (20) are substantially lower than those of other alcohols, which may result in longer ignition delays and incomplete combustion. But in a dual‐fuel system, when combined with the diesel‐biodiesel blend, their main function is to improve combustion.

#### 2.3.2 Calorific Value

An indicator of the quantity of energy released during combustion was the calorific value. In general, higher values result in better engine performance and higher power output. Diesel has the highest calorific value (42 450 kJ kg^−1^), which means that it releases the most energy per unit of fuel, resulting in higher BTE and better engine performance. In comparison to diesel, CIWFOBD has a lower calorific value, which leads to a lower power output and BTE. With a calorific value (41 336 kJ kg^−1^) that was closer to diesel than pure CIWFOBD, the 80D+20CIWFOBD blend improves performance and releases more energy. In comparison to diesel and CIWFOBD, both alcohols have lower calorific values: butanol (32 650 kJ kg^−1^) and pentanol (34 650 kJ kg^−1^). If used alone, this could lead to a lower power output. But rather than outright replacing the primary fuel's energy contribution, their function in the premixed fuel system was to enhance combustion characteristics.

#### 2.3.3 Density

Density had an impact on the injection's mass flow rate and fuel atomization. Fuels with higher densities typically require more fuel to be injected for a given volume, which can have an impact on emissions and combustion efficiency. Diesel's moderate density (821 kg m^3^) allows for effective combustion by balancing mass flow rate and atomization. Because CIWFOBD has a higher density (854 kg m^3^), more fuel mass was injected per injection. If this was not managed properly, this could result in incomplete combustion and higher particulate emissions. Compared to pure CIWFOBD, the 80D+20CIWFOBD blend's density (830 kg m^3^) was more akin to that of diesel, which improves atomization and combustion efficiency. Pentanol (816 kg m^3^) and butanol (809 kg m^3^) had lower densities, which enhance their atomization and the premixed combustion process, resulting in improved combustion and reduced emissions.

#### 2.3.4 Flash Point

The temperature at which a fuel could evaporate and turn into an ignitable mixture in the air was known as the flash point. Lower flash points signify a higher risk of pre‐ignition, whereas higher flash points suggest safer handling and storage. The flash point of diesel, which is 69 °C, permits effective vaporization during combustion while guaranteeing safe handling. Because CIWFOBD has a higher flash point (93 °C), it takes more energy to vaporize, which could result in incomplete combustion and increased emissions, especially in cold weather. Compared to pure CIWFOBD, the blend 80D+20CIWFOBD has a flash point that strikes a balance between safety and effective vaporization, resulting in superior combustion. Because of their low flash points, alcohols like butanol (36 °C) and pentanol (48 °C) can vaporize and mix with air more easily, which can increase combustion efficiency but also raise the risk of pre‐ignition if not handled carefully.

#### 2.3.5 Heat of Vaporization

A substance needs heat of vaporization to change from a liquid to a gas. Higher heat of vaporization fuels can slow combustion but also more effectively cool the intake charge, resulting in denser air‐fuel mixtures. Diesel contributes to stable combustion because of its moderate heat of vaporization (284 kJ kg^−1^), which enables effective vaporization and mixing without significantly cooling the intake charge. Cooler intake temperatures and denser air‐fuel mixtures may result from CIWFOBD's higher heat of vaporization (394 kJ kg^−1^), but slower vaporization and combustion may also result in increased emissions. The blend 80D+20CIWFOBD enhances combustion efficiency and intake charge cooling with a balanced heat of vaporization of 320 kJ kg^−1^. The exceptionally high heat of vaporization of butanol (596 kJ kg^−1^) can dramatically cool the intake charge, increasing air density but possibly delaying combustion. Pentanol performs better in the experiment because it better balances cooling and combustion efficiency due to its lower heat of vaporization (310 kJ kg^−1^).

#### 2.3.6 Oxygen Content

Fuels with higher oxygen contents burn more efficiently by supplying more oxygen to the combustion process, which can result in a more complete burn and lower emissions. Because diesel has zero oxygen content and depends only on intake air for combustion, there may be incomplete combustion in some situations. Though it still produces a lower BTE than diesel due to other factors like lower calorific value and higher density, the 14.25 percent oxygen content in CIWFOBD improves combustion and helps to reduce CO and unburned hydrocarbon emissions. Comparing the blend 80D+20CIWFOBD to pure CIWFOBD, the blend performs better and emits fewer emissions due to its moderate oxygen content (5.20%). Due to their high oxygen contents—21% for butanol and 18% for pentanol—both alcohols significantly increase combustion efficiency while lowering CO and smoke emissions. Pentanol performs better in the study because of its other qualities and slightly lower oxygen content, which produce ideal combustion characteristics.

#### 2.3.7 Viscosity

Fuel atomization during injection, fuel flow characteristics, and fuel spray pattern formation are all impacted by viscosity. Poor atomization can arise from fuels with excessive viscosity, and leaks and insufficient lubrication could be caused by fuels with insufficient viscosity. Diesel had a moderate viscosity of 2.84 cSt, which helps to promote effective combustion by ensuring good atomization and fuel spray. Because of its higher viscosity (3.51 cSt), CIWFOBD may burn unevenly and with poorer atomization, which could result in lower BTE and higher emissions. Comparing the blend 80D+20CIWFOBD to pure CIWFOBD, the viscosity of the latter was 2.97 cSt, which is closer to that of diesel, improving atomization and combustion efficiency. Low viscosities of 2.7 cSt for butanol and 2.9 cSt for pentanol, respectively, help with atomization and air mixing, which raises the efficiency of combustion. The study's observed decreases in CO and smoke emissions can be attributed to this.

The fuels' chemical characteristics greatly impact emissions and engine performance in this study. Diesel was the industry standard for clean burning and low emissions due to its higher cetane number, higher calorific value, and moderate properties. CIWFOBD had a higher oxygen content, but its lower calorific value and higher viscosity cause it to perform worse and emit more emissions. The overall qualities of diesel were enhanced by blending CIWFOBD, leading to improved emissions and performance. Despite having lower cetane numbers and calorific values, the alcohols—butanol and pentanol—contribute to better combustion efficiency because of their higher oxygen content and reduced viscosity. To prevent pre‐ignition and guarantee optimum performance, their higher heat of vaporization and lower flash points must be carefully controlled. This work indicates that when the chemical properties are balanced correctly, fuels could be blended and oxygenated substances, such as alcohols, added to improve engine performance, lower emissions, and increase combustion efficiency.

### 2.4 Engine Arrangement with Uncertainty

**Figure 3:**
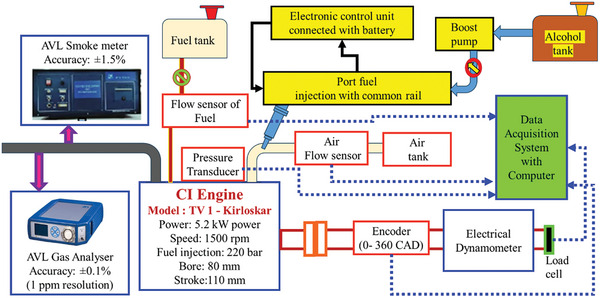
Investigational alcohol premixing engine layout.

A compression ignition (CI) engine test rig was used in the experiment to evaluate different fuel blends, including biodiesel made from the waste product of chicken intestines. Under controlled conditions, the setup was intended to measure engine performance, combustion performance, and emission characteristics. **Figure** **3** depicts the investigational alcohol premixing engine layout. This setup has a Direct injection CI engine. Their details are given in **Table** **3**. Loading is done using the eddy current method by using a load cell. The engine receives a regulated load from the eddy current dynamometer, which produces electromagnetic resistance. A conductive substance experiences eddy currents when the engine turns the dynamometer. The engine is subjected to a resistive load as a result of these currents producing an opposing magnetic field to motion. The engine's force or torque is measured by the load cell in relation to the resistance that the eddy current dynamometer provides. It transforms this mechanical force into an electrical signal so that engine performance can be measured, recorded, and examined. The crank angle encoder is a device used to measure the angle at which the crankshaft is positioned. The transducers can measure the pressure inside the cylinder. The suction pulls in clean, dust‐free air from the air tank.

**Table 3:** Detail of CI Engine Test Rig.

Description	Specification
Type	CI engine
Stroke	0.11 m
Speed	1500 rpm
Power	5.2 kW
Model	TV1
Make	Kirloskar
Injection Type	Direct
Injection Timing	23° TDC
Injection pressure	200 bar
Cooling	Water
Compression ration	17.5
Bore	0.0875 m

A fuel tank supplies fuel directly to the combustion chamber via an injector. It was called direct injection of the fuel. The flow rate of liquids could be measured using individual flow sensors. By using control knabs load was given to the engine through the dynamometer. In data collection software installed computer stores the data from the data acquisition system which was connected to all possible data collection of the system. Emission measurement particulars were mentioned in **Table** **4**.

**Table 4:** AVL – Smoke meter and gas analyzer details for Emission measurement.

Details	Smoke	CO_2_	UHC	CO	NOx
Resolution	0.10%	0.1% vol	10 ppm	0.001% vol	1 ppm
Range	0%–100%	0%–20%	0–2000 ppm	0‐15%	0–6000 ppm

CI Engine (TV 1‐Kirloskar Model), 5.2 kW of power, 1500 rpm of speed, 220 bar of fuel injection pressure, 80 mm of bore, and 110 mm of stroke Test fuels were burned in this direct injection engine. Throughout the trials, the engine's performance was observed. The fuel system comprises the fuel tank, which supplies the engine with fuel blends. The fuel consumption rate was measured using a fuel flow sensor. Accurate fuel delivery into the combustion chamber is ensured by port fuel injection with a common rail. Connected to mix and test alcohol additives are the alcohol tank and boost pump. In the System of Air Supply, the Clean air was stored in the air tank and supplied to the engine. In order to maintain a constant air‐fuel mixture, the airflow sensor measures the intake air flow rate. The measuring instruments and sensors: the pressure transducer was provided to assess combustion efficiency; this device gauges the pressure inside the cylinder during combustion. To correlate in‐cylinder pressure with crank angle, the encoder (0‐360 CAD) must keep track of the crankshaft angle. An electrical dynamometer provides the engine with loading so that performance can be measured under various load scenarios. The System of Data Acquisition was networked to a computer which gathers and stores data from multiple sensors and instruments throughout the investigation. For the analysis of performance metrics such as Brake Thermal Efficiency (BTE) and Brake Specific Fuel Consumption (BSFC), the system was essential. The AVL Smoke Meter provides information on particulate emissions by measuring smoke opacity with an accuracy of 1.5%, With an accuracy of 0.1% (1 ppm resolution), the AVL Gas Analyzer measures emissions like CO, NOx, and HC, enabling a thorough examination of exhaust gases. Conditions for the experiment were Engine Speed: 1500 rpm was the steady speed at which the engine ran. Fuel Injection Pressure was set to ensure adequate atomization and combustion, the fuel was injected at a pressure of 220 bar. Loading Mechanism was to enable controlled testing under different load conditions, the engine was loaded using an electrical dynamometer. Tested Fuel Blends Seven distinct fuel blends were tested, including neat diesel and CIWFOBD with varying butanol and pentanol percentages. With the help of this configuration, it was possible to assess the fuel blends' combustion properties, engine output, and emissions to choose the one that would work best for CI engines

In addition to that the premixing set‐up was created for the alcohol premixing. An alcohol tank was placed to hold the alcohol. A small pump was used to supply the alcohol to the common rail injector which acts as the port injector placed in the inlet manifold. The control of alcohol flow was controlled by the electronic control unit (ECU) powered by the external battery.

(1) $$\begin{array}{c} Total\;Uncertainty=\sqrt[{2}]{\begin{array}{c} {\left(U_{s}\right)}^{2}+{\left(U_{BP}\right)}^{2}+{\left(U_{CO}\right)}^{2}+{\left(U_{En}\right)}^{2}\\ +{\left(U_{W}\right)}^{2}+{\left(U_{NOx}\right)}^{2}+{\left(U_{Pr}\right)}^{2}+{\left(U_{Sp}\right)}^{2}\\ +{\left(U_{T}\right)}^{2}+{\left(U_{t}\right)}^{2}+{\left(U_{UHC}\right)}^{2} \end{array}} \end{array}$$

where,

Us: Uncertainty in the speed measurement of the engine.
U_BP_: Uncertainty in the brake power measurement.
U_CO_: Uncertainty in the measurement of carbon monoxide emissions.
U_En_: Uncertainty in the energy measurement or fuel consumption.
U_W_: Uncertainty in the weight or mass measurement.
U_NOx_: Uncertainty in the measurement of nitrogen oxide emissions.
U_Pr_: Uncertainty in the pressure measurement.
U_Sp_: Uncertainty in the specific fuel consumption measurement.
U_T_: Uncertainty in the temperature measurement.
U_t_: Uncertainty in the time measurement.
U_UHC_: Uncertainty in the unburned hydrocarbons (UHC) emissions measurement.

The data on emissions were obtained from the exhaust gas that exited from the engine's exhaust pipe. This gas was then connected to a gas analyzer and smoke meter, the specifics of which are detailed in Table 4. Any uncertainties in the measurements are described in **Table** **5**. The best way to determine the overall degree of uncertainty is to use Equation (1).^[^ ^26^ ^]^ The ±2.1795% is the total uncertainty of measurements. Employing this formula stated in Equation (1) one can ensure accuracy and consistency throughout the computation process.

**Table 5:** Particulars of Uncertainty.

Particulars of measurement	Accuracy [uncertainty %]	Uncertainty
UHC	±0.5%	0.25
Time	±0.5%	0.25
Temperature	± 1.0%	1
Speed	±1.0%	1
Pressure	± 0.5%	0.25
NOx	±1.0%	1
Load	±0.5%	0.25
Encoder	±0.5%	0.25
CO	±0.01%	0.0001
Brake power	±0.5%	0.25
AVL Smoke meter – Smoke – S	±1.0%	1
Sum		5.5
Total uncertainty		±2.34%

## 3 Results and Discussion

The following methodology was used to determine the in‐cylinder pressure, heat release rate, brake‐specific fuel consumption (BSFC), brake thermal efficiency (BTE), and emissions like CO, NOx, smoke opacity, and HC.

Using a Direct Injection (DI) 5.2 kW CI engine, seven different types of fuels were tested under the same conditions: 100D, CIWFOBD, 80D+20CIWFOBD, 80D+20CIWFOBD‐Bu10%, 80D+20CIWFOBD‐Bu20%, 80D+20CIWFOBD – Pen10%, and 80D+20CIWFOBD‐Pen. 1500 rpm was the constant speed at which the tests were performed. Using a load cell and the eddy current method, the engine was loaded. Transducers were used to measure the pressure inside the cylinder, and a crank angle encoder was used to measure the position of the crankshaft. An air tank provided clean, dust‐free air for the air intake system to draw in. Through, the use of an injector, the fuel was injected directly into the combustion chamber through a direct injection system. Individual flow sensors were used to measure the fuel's flow rate. The dynamometer's control knobs were used to apply the load to the engine. Data from multiple sensors was gathered by the data acquisition system and stored in a computer running data collection software. An AVL smoke analyzer built into the test rig was used to analyze emissions, including CO, NOx, smoke opacity, and HC. The Crank angle encoder and pressure transducer data were used to determine the in‐cylinder pressure and heat release rate, while engine output and fuel consumption measurements were used to compute BTE and BSFC.

The experimentally measured values and the calculated values from those data were explained in these results and discussed in the following Equations ((2), (3), (4)) for heat release rate, BTE, and BSFC, respectively.^[^ ^26^ ^]^ θ,  *Q*,  *v*,  *and* *P* are referred as the crank angle, heat, volume and pressure of the particular crank position, respectively. γ,  *d*θ,  *dQ*,  *dv*,  *and* *dp*, is the adiabatic index, change in crank angle degree, change in heat, volume change, and change in pressure respectively.

(2) $$\begin{array}{cc} & Heat\;release\;rate=\frac{dQ}{d\theta }\\ & \;=\left\{\left(\frac{\gamma }{\gamma -1}\right)p\left(\frac{dv}{d\theta }\right)\right\}+\left\{\left(\frac{1}{\gamma -1}\right)v\left(\frac{dp}{d\theta }\right)\right\} \end{array}$$

(3) $$Brake\;thermal\;efficiency=\frac{Brake\;power\;in\;kW}{Total\;heat\;input\;in\;kW\;}\times \;100$$

(4) $$\begin{array}{cc} & Brake\;specific\;fuel\;consumption\\ & \;=\frac{Total\;fuel\;consumption\;of\;main\;fuel\;in\frac{kg}{s}\times \;3600}{Brake\;power\;in\;kW} \end{array}$$

**Figure 4:**
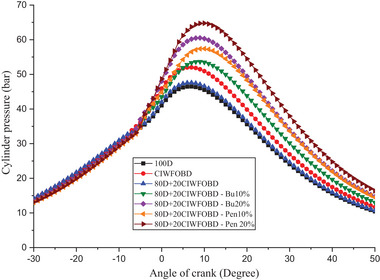
Influence of alcohol premixing with CIWFOBD blend in‐cylinder pressure.

The influence of alcohol premixing with CIWFOBD blend in‐cylinder pressure is explained in **Figure** **4**. The 100D, CIWFOBD, 80D+20CIWFOBD, 80D+20CIWFOBD – Bu10%, 80D+20CIWFOBD – Bu20%, 80D+20CIWFOBD – Pen10%, and 80D+20CIWFOBD – Pen 20% has 46.47 bar, 52.01 bar, 47.58 bar, 53.66 bar, 60.52 bar, 57.42 bar, and 64.76 bar of peak cylinder pressure at peak load condition respectively, Compared to sole CIWFOBD, 80D+20CIWFOBD blend fuel have reduced peak pressure which is nearly to the diesel operation this reduction because of reduction on the viscosity, and increase of the heating value of the 80D+20CIWFOBD through the 80% of diesel involvement on the CIWFOBD.^[^ ^17^, ^20^ ^]^

In butanol and pentanol premixing 20% have produced higher peak pressure than 10%. The 80D+20CIWFOBD – Bu20% produced 16% and 30.23% higher peak pressure than CIWFOBD and diesel. This is because of the increased vaporization of the butanol during the two stages before the combustion it produces a premixed charge to make better combustion with higher ignition delay.^[^ ^24^, ^27^, ^28^ ^]^

But the highest peak pressure is achieved by the 80D+20CIWFOBD – Pen 20% even 24.50% and 35.15% higher than CIWFOBD and diesel. It produces an even better homogeneous mixture formation of the charge with a higher ignition delay than butanol premixing. Also, the higher heating value of the pentanol supports the improved mixture formation than the butanol at 20% of premixing.^[^ ^25^, ^29^, ^30^ ^]^

**Figure 5:**
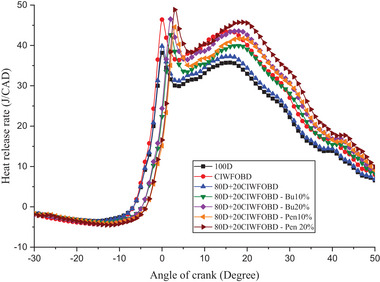
Influence of alcohol premixing with CIWFOBD blend in heat release rate.

Encouragement of alcohol premixing with CIWFOBD blend in HRR is explained in **Figure** **5**. The 100D, CIWFOBD, 80D+20CIWFOBD, 80D+20CIWFOBD‐Bu10%, 80D+20CIWFOBD – Bu20%, 80D+20CIWFOBD – Pen10%, and 80D+20CIWFOBD – Pen 20% has 38.16 J/CAD, 46.40 J/CAD, 39.81 J/CAD, 42.64 J/CAD, 46.53 J/CAD, 44.56 J/CAD, and 48.86 J/CAD of maximum HRR at peak load condition respectively, Compared to CIWFOBD, 80D+20CIWFOBD blend fuel have reduced HRR which is higher than diesel operation this decrease on account of the decrease on the viscosity, and rise of the heating value and cetane number through the 80% of diesel involvement on the CIWFOBD.^[^ ^17^, ^20^ ^]^

In butanol and pentanol premixing 20% have produced higher HRR than 10%. The 80D+20CIWFOBD – Bu20% produced 21.94% and 0.28% higher HRR than CIWFOBD and diesel. During the two stages before combustion, butanol undergoes increased vaporization. This results in the production of a premixed charge that enables better combustion with a higher ignition delay. The increased vaporization of butanol enhances the combustion process by facilitating the even distribution of fuel and air in the combustion chamber.^[^ ^24^, ^27^, ^28^ ^]^

But the highest HRR is achieved by the 80D+20CIWFOBD – Pen 20% which is 23.60% and 5.29% higher than CIWFOBD and diesel. It produces an even better homogeneous mixture formation of the charge with a higher ignition delay than butanol premixing. Also, the higher heating value of the pentanol supports the improved mixture formation than the butanol at 20% of premixing. The increased heating value of the charge increases the heat release rate of the combustion.^[^ ^25^, ^29^, ^30^ ^]^

**Figure 6:**
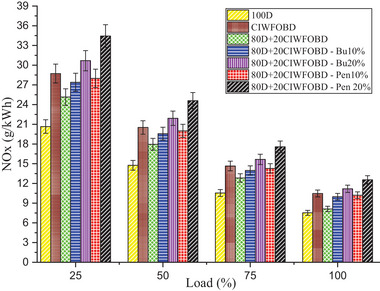
Influence of alcohol premixing with CIWFOBD blend in NOx.

Encouragement of alcohol premixing with CIWFOBD blend in NOx emission is enlightened in **Figure** **6**. The 100D, CIWFOBD, 80D+20CIWFOBD, 80D+20CIWFOBD – Bu10%, 80D+20CIWFOBD – Bu20%, 80D+20CIWFOBD – Pen10%, and 80D+20CIWFOBD – Pen 20% has 7.53, 10.47, 8.12, 9.98, 11.18, 10.20, and 12.55 g kWh^−1^ of NOx emission at peak load condition respectively, Compared to CIWFOBD, 80D+20CIWFOBD blend fuel have 22.46% reduced NOx emission which is 7.81% higher than diesel operation this reduction in consequence of the decrease on the cylinder pressure with the heat release rate of the blend than the CIWFOBD. This pressure and heat release rate change reduction supports the reduction of NOx emission than CIWFOBD.

In butanol and pentanol premixing 20% have produced higher NOx emission than 10%. The highest NOx emission is produced by 80D+20CIWFOBD – Bu20% produced at 48.51%, and 6.82% higher NOx emission than CIWFOBD and diesel. Increased in‐cylinder pressure concerning the butanol premixing increases the cylinder temperature and the available oxygen in the butanol increases the possible oxidation with the higher reaction temperature producing the increased NOx emission.^[^ ^24^, ^27^, ^28^ ^]^

Also, the 80D+20CIWFOBD – Pen is 20% even 47.96%, and 19.89% higher than CIWFOBD and diesel. It produces higher NOx because of the improved oxidation with the oxygen content available on the pentanol. The increased pressure and higher heat release rate simultaneously support the increase in the temperature of the cylinder. This increase in cylinder temperature creates an increase in the NOx emission with temperature. Also, the better combustion with butanol premixing produced slightly lesser NOx emission than the butanol premixed condition with their higher heating value of the charge than the butanol charge.^[^ ^25^, ^29^, ^30^ ^]^

Considering the environmental and regulatory restrictions on NOx emissions, the higher NOx emissions observed with some fuel blends, such as the 80D+20CIWFOBD – Bu20% and 80D+20CIWFOBD – Pen20%, are worrisome. While there are many strategies for reducing NOx emissions, this study recommends passive strategies. When combined with these biodiesel blends, EGR might significantly decrease NOx emissions without compromising engine performance. In order to promote more thorough combustion or modify the combustion characteristics to lower peak temperatures, NOx‐reducing additives can be blended in with the fuel. Using specific additives that are compatible with biodiesel and alcohol blends could be an effective strategy to mitigate NOx emissions without sacrificing performance.

**Figure 7:**
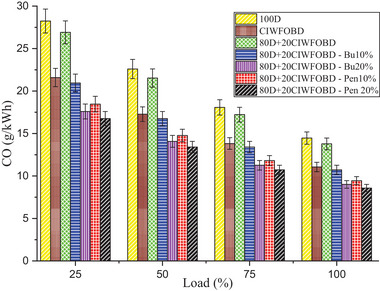
Influence of alcohol premixing with CIWFOBD blend in CO emission.

Encouragement of alcohol premixing with CIWFOBD blend in CO emission is enlightened in **Figure** **7**. The 100D, CIWFOBD, 80D+20CIWFOBD, 80D+20CIWFOBD – Bu10%, 80D+20CIWFOBD – Bu20%, 80D+20CIWFOBD – Pen10%, and 80D+20CIWFOBD – Pen 20% has 14.46, 11.06, 13.78, 10.73, 9.01, 9.45, and 8.58 g kWh^−1^ of CO emission at highest load condition respectively, Compared to CIWFOBD, 80D+20CIWFOBD blend fuel have 24.63% reduced CO emission which is 4.71% lesser than diesel. The incomplete combustion formation during the combustion produces the major amount of CO emission. The viscosity variation and lesser vaporization quality with higher density cause the incomplete combustion of the blend.^[^ ^1^, ^31^ ^]^

In butanol and pentanol premixing 20% have produced lesser CO emission than 10%. The lesser CO emission is produced by 80D+20CIWFOBD – Bu20% which is 18.51% and 37.69% lesser CO emission than CIWFOBD and diesel. The butanol premixing provides the excess oxygen content to the charge to make improved combustion. This improved combustion produced the maximum possible burning of the charge with lesser final combustion product such as lesser CO emission. Also, the heat release rate supports the higher possibility of the CO into the CO_2_ conversion which will also reduce the CO emission.^[^ ^24^, ^27^, ^28^ ^]^

Further CO emission reduction was achieved, and the least CO emission produced by the 80D+20CIWFOBD – Pen was 20% even 22.39%, and 53.18% less than CIWFOBD and diesel. The higher heating value of the pentanol and their higher latent heat of evaporation support the higher heat release rate with excess oxygen availability produced by the pentanol in the charge. This highest heat release rate and improved charge quality by the pentanol premixing produced a higher amount of CO to CO_2_ conversion. This will lead to the lesser CO emission which is nearly half of the diesel CO emission.^[^ ^25^, ^29^, ^30^ ^]^

**Figure 8:**
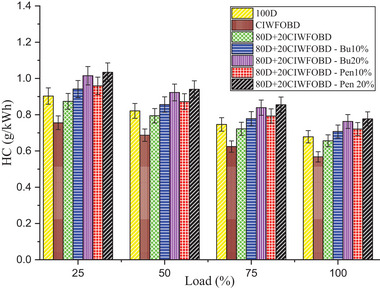
Influence of alcohol premixing with CIWFOBD blend in HC emission.

Encouragement of alcohol premixing with CIWFOBD blend in unburnt hydrocarbon emission is enlightened in **Figure** **8**. The 100D, CIWFOBD, 80D+20CIWFOBD, 80D+20CIWFOBD – Bu10%, 80D+20CIWFOBD – Bu20%, 80D+20CIWFOBD – Pen10%, and 80D+20CIWFOBD – Pen 20% has 0.679, 0.568, 0.66, 0.708, 0.763, 0.721, and 0.777 g kWh^−1^ of HC emission at highest load condition respectively. Compared to CIWFOBD, 80D+20CIWFOBD blend fuel has a 15.60% increased HC emission which is 3.26% less than diesel. The unburnt HC is produced due to the incomplete combustion formation during the combustion. This increase is because the viscosity variation and lesser vaporization quality with higher density cause the incomplete combustion of the blend.^[^ ^1^, ^8^, ^18^ ^]^

In butanol and pentanol premixing 20% have produced higher unburnt HC emissions than 10%. The higher HC emission is produced by 80D+20CIWFOBD – Bu20% which is 34.34% and 12.42% lesser HC emission than CIWFOBD and diesel. The butanol premixing provides the possibility of increased hydrocarbon participation in the charge. The quenching formation produced by the butanol during premixing in suction itself cools the available exhaust gasses in the chamber which are stuck in the surface of the cylinder. This will get excited after combustion in the exhaust stroke. This will increase the HC emission.^[^ ^24^, ^27^, ^28^ ^]^

Similarly, further unburnt HC emission increased in 80D+20CIWFOBD – Pen 20% even 36.84%, and 17.34% higher than CIWFOBD and diesel. It is the highest HC emission in the remaining test conditions. This increase is because of higher hydrocarbon content available than butanol premixing. The quenching created by the pentanol premixing provides a higher unburnt HC. Also, during the combustion, the unburnt fuel content having the hydrocarbon was sent out as the unburnt HC emission.^[^ ^25^, ^29^, ^30^ ^]^

**Figure 9:**
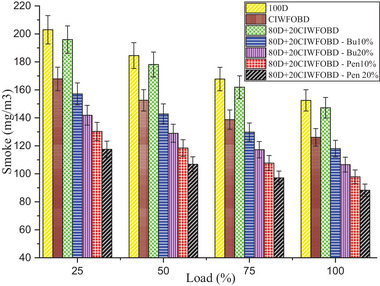
Influence of alcohol premixing with CIWFOBD blend in smoke emission.

Encouragement of alcohol premixing with CIWFOBD blend in smoke emission is enlightened in **Figure** **9**. The 100D, CIWFOBD, 80D+20CIWFOBD, 80D+20CIWFOBD – Bu10%, 80D+20CIWFOBD – Bu20%, 80D+20CIWFOBD – Pen10%, and 80D+20CIWFOBD – Pen 20% has 152.5, 126.1, 147.22, 118.0, 106.6, 97.9, and 88.3 mg m^3^ of smoke emission at peak load condition respectively. Compared to CIWFOBD, 80D+20CIWFOBD blend fuel has 16.75% increased smoke emission which is 3.46% lesser than diesel operation this increase in smoke is because of the diesel participation with CIWFOBD but the 20% CIWFOBD participation reduces the diesel smoke. This variation is due to the viscosity variation and higher latent heat of evaporation rate of the blend.^[^ ^32^, ^33^ ^]^

In butanol and pentanol premixing 20% have produced lesser smoke emission than 10%. The lesser smoke emission is produced by 80D+20CIWFOBD – Bu20% produced 15.46%, and 30.10% lesser smoke emission than CIWFOBD and diesel. Increased in‐cylinder pressure with respect to the butanol premixing increases the cylinder temperature and the available oxygen in the butanol increases the possible oxidation with the higher reaction temperature producing a reduction in the smoke formation.^[^ ^24^, ^27^, ^28^ ^]^

Also, the 80D+20CIWFOBD – Pen is 20% even 29.98%, and 50.92% higher smoke emission than CIWFOBD and diesel. It produces less smoke because of the improved oxidation with the oxygen content available on the pentanol. The premixing produced a higher possibility of the homogeneous mixture formation of the charge with butanol. It led to butter combustion with increased pressure and higher heat release rate simultaneously supporting smoke reduction. The conversion of the sources of the soot particles was burned and produced the heat energy during the combustion.^[^ ^25^, ^29^, ^30^ ^]^

**Figure 10:**
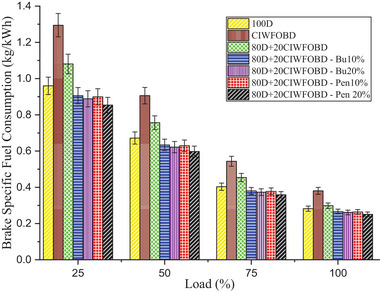
Influence of alcohol premixing with CIWFOBD blend in BSFC.

Variation of alcohol premixing with CIWFOBD blend in brake‐specific fuel consumption is described in **Figure** **10**. The 100D, CIWFOBD, 80D+20CIWFOBD, 80D+20CIWFOBD – Bu10%, 80D+20CIWFOBD – Bu20%, 80D+20CIWFOBD – Pen10%, and 80D+20CIWFOBD – Pen 20% has 0.282, 0.381, 0.298, 0.266, 0.261, 0.264, and 0.251 kg kWh^−1^ of BSFC peak load condition respectively. Compared to CIWFOBD, 80D+20CIWFOBD blend fuel has 21.55% lesser BSFC which is 5.8% higher than diesel operation this variation is on account of the viscosity reduction, and increased cetane number and heating value of the blend through the 80% of diesel involvement on the CIWFOBD at the same time compared to diesel the increase in the BSFC is due to the participation of the 20% biodiesel which have to supply excess fuel to produce equal power.^[^ ^19^, ^34^ ^]^

In butanol and pentanol premixing 20% have produced lesser specific fuel consumption than 10%. At full load, the 80D+20CIWFOBD – Bu20% produced 31.36% and 7.42% less BSFC than CIWFOBD and diesel. The participation of the butanol increases the charge quality through premixing so the required main fuel contribution for the combustion. The amount of the main fuel required is to produce equal power in peak load.^[^ ^24^, ^28^ ^]^

On the other hand, the least BSFC is achieved by the 80D+20CIWFOBD – Pen 20% which is 34.06% and 8.21% less than CIWFOBD and diesel. Due to the higher calorific value and latent heat of evaporation of pentanol, 80D+20CIWFOBD – Pen 20% produces even improved homogeneous mixture formation than butanol premixing. This will reduce the need for the amount of the participation of the main fuel such as 80D+20CIWFOBD which is injected inside the cylinder.^[^ ^28^, ^30^ ^]^

**Figure 11:**
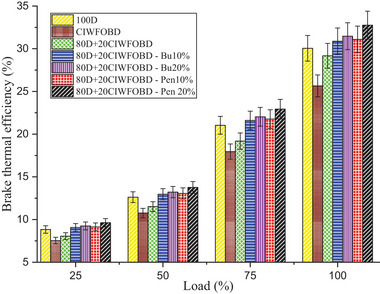
Influence of alcohol premixing with CIWFOBD blend in BTE.

Variation of alcohol premixing with CIWFOBD blend in BTE is described in **Figure** **11**. The 100D, CIWFOBD, 80D+20CIWFOBD, 80D+20CIWFOBD – Bu10%, 80D+20CIWFOBD – Bu20%, 80D+20CIWFOBD – Pen10%, and 80D+20CIWFOBD – Pen 20% has 30.05%, 25.65%, 29.17%, 30.88%, 31.47% 31.10%, and 32.76% of BTE at peak load condition respectively. Compared to CIWFOBD, 80D+20CIWFOBD blend fuel has 13.73% increased BTE which is 2.93% lesser than diesel operation this variation is on account of the decrease in the viscosity, and rise of the heating value and cetane number through the 80% of diesel involvement on the CIWFOBD.^[^ ^19^, ^34^ ^]^

In butanol and pentanol premixing 20% have produced higher brake thermal efficiency than 10%. The 80D+20CIWFOBD – Bu20% produced 22.70% and 4.73% higher HRR than CIWFOBD and diesel. At the two stages before combustion, butanol experiences augmented vaporization with better mixing and higher latent heat of evaporation. This consequences in the production of a premixed charge that enables better combustion with a higher ignition delay. The even distribution of fuel and air in the combustion chamber is facilitated by the increased vaporization of butanol, thus improving the combustion process.^[^ ^24^, ^27^, ^28^ ^]^

The maximum BTE is achieved by the 80D+20CIWFOBD – Pen 20% which is 27.73% and 10.57% higher than CIWFOBD and diesel. 80D+20CIWFOBD – Pen 20% produces even improved homogeneous mixture formation of the charge with a higher ignition delay than butanol premixing. Also, the higher heating value of the pentanol provides an improved mixture formation than the butanol at 20% of premixing. The increased heating value of the charge increases the heat release rate of the combustion. A higher heating value of the charge increases the BTE.^[^ ^28^, ^29^, ^30^, ^35^ ^]^

Social Implications of this study include the following. This study offers a useful method for handling the waste from chicken intestines, which is usually thrown away and pollutes the environment. The study encourages waste‐to‐energy conversion by turning waste into biodiesel, which lessens the load on landfills and lowers greenhouse gas emissions. The production of biodiesel from chicken intestine waste gives the poultry industry an extra source of income. It turns a byproduct that would otherwise be thrown away into a useful good, which could reduce energy costs and boost regional economies. By lowering reliance on fossil fuels, developing alternative fuel sources like CIWFOB improves energy security. In addition to ensuring a more sustainable energy supply, this diversification of energy sources can stabilize fuel prices. Reducing emissions from engine exhausts, such as smoke and carbon monoxide (CO), can enhance public health and the quality of the air. This is particularly crucial in cities where air pollution is a major issue.

The details od SDG attainments are as follows. The study supports the creation of sustainable and alternative energy sources, which is in line with SDG 7. The study helps to ensure that everyone has access to modern, affordable, sustainable, and reliable energy by blending CIWFOB with diesel and optimizing engine performance. The biodiesel production process encourages ethical consumption and production methods. In order to support sustainable economic growth, it highlights how crucial it is to reduce waste and use resources effectively (SDG 12). Climate‐Related Action: Through lowering greenhouse gas emissions and encouraging cleaner engine combustion, the study advances SDG 13. It emphasizes how crucial innovation is to reducing transportation's carbon footprint and addressing climate change. Infrastructure, Industry, and Innovation: Innovation in the fuel and automotive industries is encouraged by this investigation. Through the incorporation of fuels derived from waste, facilitates the creation of sustainable infrastructure and encourages equitable and sustainable industrialization (SDG 9).

## 4 Conclusion 

The main energy source used in this investigation is biodiesel made from chicken intestinal waste fat oil to run diesel engines. Diesel is mixed with this fuel to improve engine characteristics, butanol and pentanol were also investigated under premixed form in different percentage energetic contributions for this blend. The following findings are the result of this investigation.

- It was found that the 5.2 kW CI engine can run on chicken intestine waste fat oil biodiesel (CIWFOBD), which withstands all loading conditions but performs less well than diesel.
- It is also feasible to run the CI engine in null to full load condition with an 80D+20CIWFOBD blend, which has a slightly higher BTE than CIWFOBD but less than diesel and higher emissions than diesel.
- To run the CI engine, butanol, and pentanol can be premixed separately while using the 80D+20CIWFOBD blend.
- When combining the aforementioned alcohols with an 80D+20CIWFOBD blend, a 20% energy share yielded better results than a 10% energy contribution.
- Because of the enhanced combustion brought about by the premixing with higher heating value, the 20% butanol premixed 80D+20CIWFOBD blend has produced 31.47% of BTE, which is 4.73%, 22.7% higher than diesel and CIWFOBD. 9.01 g kWh^−1^ of CO and 106.6 mg m^3^ of smoke emissions are 37.69% and 30.1% lower than diesel and 18.51% and 15.46% lower than CIWFOBD respectively. This is because butanol produces a more homogenous mixture and oxidizes at a higher rate with the oxygen that is available.
- Using a 20% pentanol premixed 80D+20CIWFOBD blend, 32.76% of BTE was produced, which is 10.57% and 27.73% more than diesel and CIWFOBD. This is because the higher heating value content of the pentanol premixing improves combustion.
- 8.58 g kWh^−1^ of CO and 88.3 mg m^3^ of smoke emission are respectively 55.18% and 50.92% less than diesel and 22.39% and 29.98% less than CIWFOBD. This is because butanol burns more efficiently and forms a nearly homogenous mixture with higher oxidation thanks to the available oxygen.
- 12.55 g kWh^−1^ of NOx emissions, which is more than diesel and CIWFOBD due to the higher HRR and in‐cylinder pressure that raise cylinder temperature. Ultimately, compared to all test conditions taken into consideration in the study, the 20% pentanol premixed 80D+20CIWFOBD blend produced an improved performance with less CO and smoke emission and slightly increased NOx emission. Therefore, it is advised to use this method rather than diesel to run the engine more efficiently.

## 5 Limitations and Future Scope

With 10% and 20% butanol and pentanol serving as port fuels, the study primarily focuses on 80% diesel and 20% CIWFOB. The results might not be as applicable to other blend ratios or other possible biofuels or additives because they were not investigated. The impact of alcohol premixing and CIWFOB on engine wear and durability over the long run is not addressed in this study. The problem of NOx emission with butanol and pentanol premixing with chicken intestine waste fat oil biodiesel is investigated in this study, and it may be possible to mitigate it through post‐treatment procedures in future research. Moreover, the blend incorporates nanoparticles to lower NOx emissions.

## Conflict of Interest

The authors declare no conflict of interest.

## Data Availability

The data that support the findings of this study are available from the corresponding author upon reasonable request.
